# Transient Increase in Zn^2+^ in Hippocampal CA1 Pyramidal Neurons Causes Reversible Memory Deficit

**DOI:** 10.1371/journal.pone.0028615

**Published:** 2011-12-07

**Authors:** Atsushi Takeda, Shunsuke Takada, Masatoshi Nakamura, Miki Suzuki, Haruna Tamano, Masaki Ando, Naoto Oku

**Affiliations:** Department of Medical Biochemistry, School of Pharmaceutical Sciences, University of Shizuoka, Global COE-21, Suruga-ku, Shizuoka, Japan; Case Western Reserve University, United States of America

## Abstract

The translocation of synaptic Zn^2+^ to the cytosolic compartment has been studied to understand Zn^2+^ neurotoxicity in neurological diseases. However, it is unknown whether the moderate increase in Zn^2+^ in the cytosolic compartment affects memory processing in the hippocampus. In the present study, the moderate increase in cytosolic Zn^2+^ in the hippocampus was induced with clioquinol (CQ), a zinc ionophore. Zn^2+^ delivery by Zn-CQ transiently attenuated CA1 long-term potentiation (LTP) in hippocampal slices prepared 2 h after i.p. injection of Zn-CQ into rats, when intracellular Zn^2+^ levels was transiently increased in the CA1 pyramidal cell layer, followed by object recognition memory deficit. Object recognition memory was transiently impaired 30 min after injection of ZnCl_2_ into the CA1, but not after injection into the dentate gyrus that did not significantly increase intracellular Zn^2+^ in the granule cell layer of the dentate gyrus. Object recognition memory deficit may be linked to the preferential increase in Zn^2+^ and/or the preferential vulnerability to Zn^2+^ in CA1 pyramidal neurons. In the case of the cytosolic increase in endogenous Zn^2+^ in the CA1 induced by 100 mM KCl, furthermore, object recognition memory was also transiently impaired, while ameliorated by co-injection of CaEDTA to block the increase in cytosolic Zn^2+^. The present study indicates that the transient increase in cytosolic Zn^2+^ in CA1 pyramidal neurons reversibly impairs object recognition memory.

## Introduction

The hippocampus plays an important role in learning, memory and recognition of novelty [1]. Recognition memory confers the ability to discriminate between novel and familiar entities. Neuropsychological analysis of amnesic patients as well as lesion experiments with non-human primates and rodents indicate that the functional integrity of the temporal lobe is essential for encoding, storage, and expression of this type of memory [2], [3]. However, it is still not clear which temporal lobe structures are directly involved in the consolidation and retrieval of object recognition memory. Particularly the role of hippocampus in these processes remains controversial. Early animal studies suggest that hippocampus is essential for object recognition memory [4], [5], [6], whereas it has been reported that hippocampal lesions do not affect object recognition memory [7], [8]. The cause for these discrepancies is not clear. However, it must be taken into account that a major drawback of these studies was that, as a result of using irreversible lesions inflicted either before or after training, they could not discriminate among the different phases of memory processing or easily exclude non-specific behavioral and physiological effects [9].

The hippocampus receives major input from the entorhinal cortex via the perforant pathway. The dentate granule cells project to the CA3 pyramidal cells via the mossy fibers. The CA3 pyramidal cells project to the CA1 pyramidal cells via the Schaffer collaterals. The three pathways are glutamatergic and terminals of them are stained by Timm's sulfide-silver method, which detects histochemically reactive (chelatable) zinc [10], [11], [12]. The zinc (Zn^2+^) predominantly exists in the presynaptic vesicles, is co-released with glutamate from zincergic neuron terminals, and serves as an endogeneous neuromodulator [13], [14]. Zn^2+^ multi-functionally modulates the induction of hippocampal long-term potentiation (LTP), a widely studied model of memory; Zn^2+^ attenuates mossy fiber LTP at low micromolar concentrations [15], [16], while potentiating NMDA receptor-dependent CA1 LTP [17], unlike NMDA receptor-independent CA1 LTP [18]. However, the role of endogenous Zn^2+^ in memory processing is poorly understood. Furthermore, much more attention has been given to Zn^2+^ neurotoxicity in neurological diseases than to the importance of Zn^2+^ homeostasis in memory processing [19], [20], [21], [22], [23], [24]. Thus, it is necessary to study the relationship between altered Zn^2+^ homeostasis and memory processing, because excitation of glutamatergic (zincergic) neurons may readily alter Zn^2+^ homeostasis under stressful condition [25], [26].

Clioquinol (5-chloro-7-iodo-8-hydroxyquinoline; CQ) forms lipophilic chelates with cations such as Zn^2+^ and Cu^2+^ and has a relatively weak affinity for zinc (K_d_, approximately 1×10^−7^ M). CQ transiently decreases Zn^2+^ levels without interfering with the tightly bound zinc pool, such as zinc fingers and numerous catalytic enzymes, which are essential for cellular functions [27], [28], [29]. Acute exposure to CQ affects object recognition memory 24 h after training, suggesting that transient lack of Zn^2+^ is involved in object recognition memory deficit [30]. In contrast, CQ also serves as an ionophore for zinc [31], [32] and is a useful tool to increase intracellular Zn^2+^. It is possible that Zn^2+^ has bidirectional actions in memory processing; appropriate increase in cytosolic Zn^2+^ might be necessary for memory processing, whereas moderate increase in cytosolic Zn^2+^ might affect it. However, it is unknown whether the moderate increase in Zn^2+^ in the cytosolic compartment affects memory processing in the hippocampus. In the present study, the moderate increase in cytosolic Zn^2+^ in the hippocampus was induced with clioquinol (CQ), a zinc ionophore. The effect of the increase in cytosolic Zn^2+^ after Zn-CQ administration was examined focused on the involvement of synaptic function of the hippocampal CA1 in object recognition memory.

## Results

### Transient increase in intracellular Zn^2+^, attenuated LTP and memory deficit

**Figure 1:**
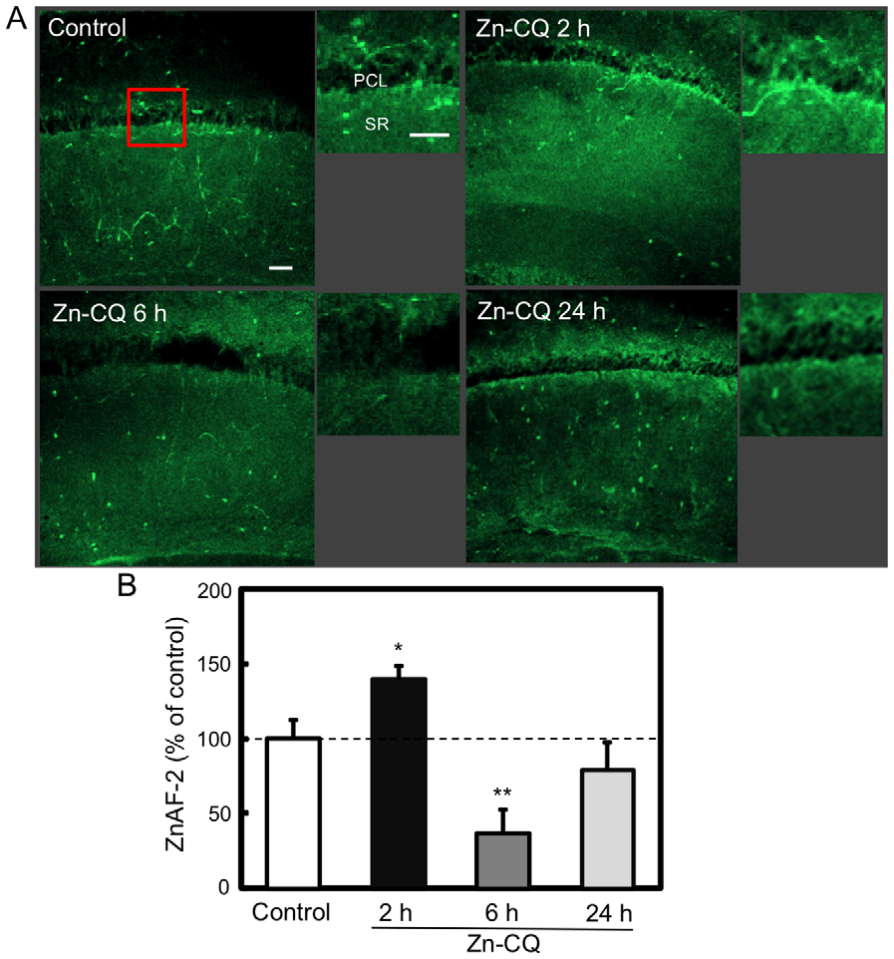
Transient increase in intracellular Zn^2+^ in the CA1 by delivery with Zn-CQ. (A) Hippocampal slices were prepared 2 (n = 7), 6 (n = 12) and 24 h (n = 7) after i.p. injection of Zn-CQ (9.8 µmol/kg) in vehicle and stained with ZnAF-2DA. Hippocampal slices prepared 2 h after vehicle injection were used as the control (n = 14). Each right panel shows the magnified CA1 area that is shown by a representative red square. Bars; 50 µm. PLC, pyramidal cell layer; SR, stratum radiatum. (B). Each bar and line (mean±SEM) represents the rate (%) of the intensity of ZnAF-2 fluorescence after Zn-CQ injection to that after vehicle injection, which was represented as 100%. *, p<0.05, **, p<0.01, vs. control (vehicle).

To check the intracellular increase in Zn^2+^ delivered with Zn-CQ, hippocampal slices were stained with ZnAF-2DA after i.p. injection of Zn-CQ (9.8 µmol/kg) (Fig. 1A). Intracellular level of Zn^2+^ measured by ZnAF-2 was significantly increased in the CA1 pyramidal cell layer (control, 100.0±12.3%; Zn-CQ 2 h, 140.0±8.6%; p<0.05 vs. control) (Fig. 1A and B). In contrast, the intracellular level of Zn^2+^ was significantly decreased in the CA1 pyramidal cell layer 6 h after Zn-CQ injection and then returned close to the control level 24 h after Zn-CQ injection (Zn-CQ 6 h, 36.6±15.8%; p<0.05 vs. control; Zn-CQ 24 h, 79.0±18.1%).

**Figure 2:**
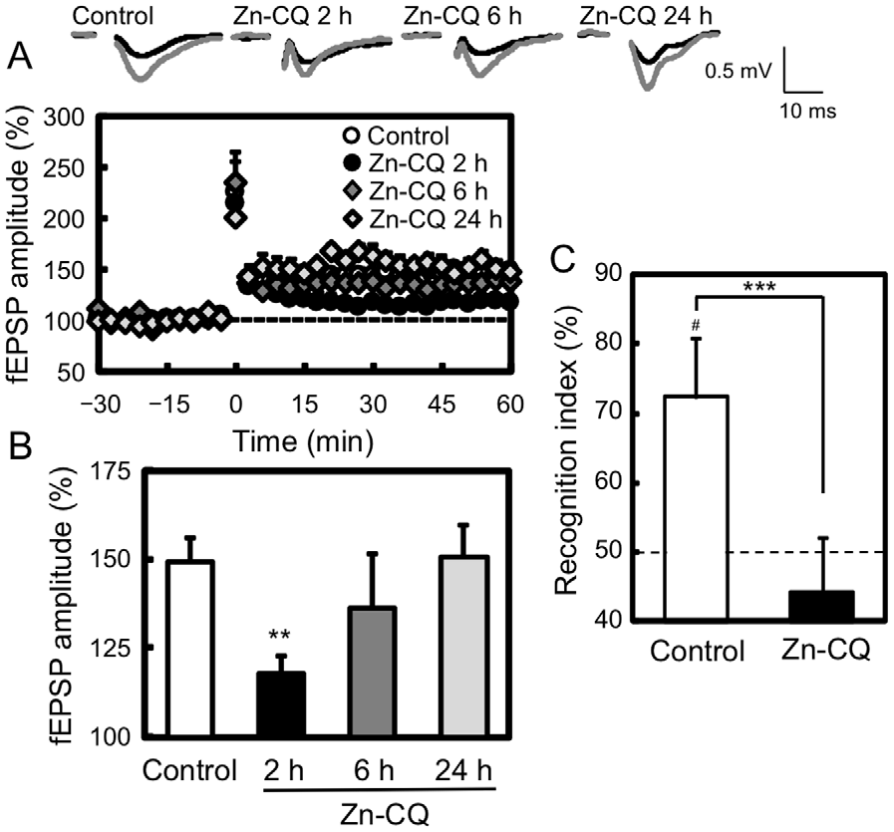
Transient attenuation of CA1 LTP and object recognition memory deficit. Hippocampal slices were prepared from the rats 2 (n = 6), 6 (n = 7) and 24 h (n = 7) after i.p. injection of Zn-CQ (9.8 µmol/kg) in vehicle. Hippocampal slices prepared 2 h after vehicle injection were used as the control (n = 15). (A) Hippocampal slices were perfused with ACSF for 60 min, tetanized at 100 Hz for 1 s, and perfused with ACSF for 60 min. Tetanic stimulation was delivered at time 0 min. Each point and line represents the mean ± SEM. (B) Each bar and line (mean ± SEM) represents the averaged fEPSP amplitude of the last 15 min (time 45–60 min). Representative fEPSP recordings at time −20 and 50 min are shown as insets (upper side). Calibration; 0.5 mV, 10 ms. **, p<0.01 vs. control. (C) Rats were placed for 10 min into an open field (n = 6). Twenty-four hours after open field exploration, rats were i.p. injected with vehicle (control) or Zn-CQ (9.8 µmol/kg) in vehicle. Two hours after injection, rats were subjected to a novel object recognition task. Each bar and line represent the mean ± SEM. ^#^, p<0.05, vs. training; ***, p<0.001, vs. control.

To check the effect of the increase in intracellular Zn^2+^ on hippocampal LTP induction, hippocampal slices were prepared after Zn-CQ injection. CA1 LTP was significantly attenuated in hippocampal slices prepared 2 h after Zn-CQ injection (control, 149.3±6.5%; Zn-CQ 2 h, 111.7±5.1%; p<0.01 vs. control), but not that prepared 6 h and 24 h after Zn-CQ injection (Zn-CQ 6 h, 136.3±14.9%; Zn-CQ 24 h, 150.7±8.6%) (Fig. 2A and B).

When the training of object recognition was performed 2 h after Zn-CQ injection, there was no significant difference in exploratory time between the control and Zn-CQ-administered rats in the training trial. The time spent to explore the two identical objects was 50.9±6.8 s and 67.4±26.2 s in the control and Zn-CQ-administered rats, respectively. The averaged recognition indices were 49.7±4.9% in the control rats and 53.6±4.2% in Zn-CQ-administered rats. The recognition index in the test trial is significantly higher than in the training trial if rats remember the familiar object. The control rats showed normal recognition memory 1 h after the training (72.3±8.6%, p<0.05 vs. training) (Fig. 2C). In contrast, Zn-CQ-administered rats showed recognition memory deficit (44.3±7.8%, p<0.05 vs. control).

### Memory deficit by increase in intracellular Zn^2+^ in the CA1

**Figure 3:**
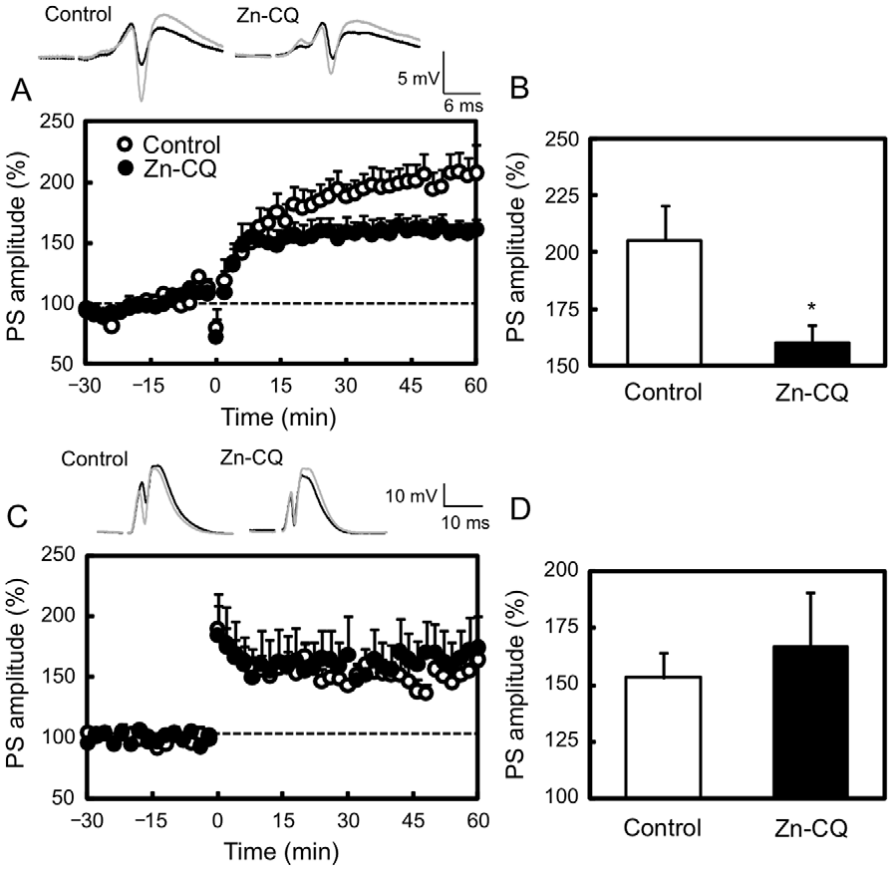
Zn-CQ injection attenuates in vivo CA1 LTP, but not DG LTP. To deliver high-frequency stimulation (HFS, 10 trains of 20 pulses at 200 Hz separated by 1 s) 2 h after i.p. injection of vehicle or Zn-CQ (9.8 µmol/kg) in vehicle, test stimuli (0.05 Hz) were delivered to the Schaffer collateral/commissural pathway or the perforant pathway of the anesthetized rats and population spike (PS) amplitudes were recorded in the CA1 (A and B) and dentate gyrus (C and D) (n = 5–6). HFS was delivered at time 0 min. Each point and line (the mean ± SEM) shows the mean of 120 s (6 points). Each bar and line (mean ± SEM) represents the averaged PS amplitude of the last 10 min (time 50–60 min). *, p<0.05, vs. control. Representative PS recordings at time −10 and 50 min are shown in the upper side.

The effect of the transient increase in intracellular Zn^2+^ was evaluated by in vivo LTP recording, because LTP induction can be exactly performed in the transient increase. When CA1 LTP was induced 2 h after Zn-CQ injection, it was significantly attenuated, in agreement with in vitro recording (control, 205.5±14.9%; Zn-CQ, 160.3±5.5%; p<0.05 vs. control) (Fig. 3A). On the other hand, dentate gryus LTP was not attenuated 2 h after Zn-CQ injection (control, 153.5±10.5%; Zn-CQ, 167.0±23.8%) (Fig. 3B).

**Figure 4:**
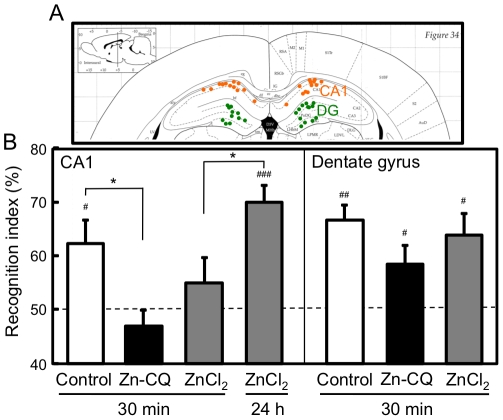
Object recognition memory after injection of Zn-CQ and ZnCl_2_ into the hippocampus. Rats were placed for 10 min into an open field. Twenty-four hours after the open field exploration, vehicle (control), Zn-CQ (9.8 nmol/rat) in vehicle, and ZnCl_2_ (9.8 nmol/rat) in vehicle were bilaterally injected via injection cannulae into the hippocampal CA1 or the dentate gyrus of the rats at the rate of 0.5 µl/min for 1 min (n = 8–9). Thirty minutes after injection, rats were trained for 5 min and then tested for 3 min in the novel object recognition task 1 h after training. In the case of ZnCl_2_ injection into the CA1, 24 h after injection, rats were subjected to the test in the same manner. (A) The dots in the brain map show the representative points injected in the CA1 (orange) and the dentate gyrus (green). (B) Each bar and line represent the mean ± SEM. ^#^, p<0.05, ^##^, p<0.01, ^###^, p<0.001, vs. training; *, p<0.05, control vs. Zn-CQ and ZnCl_2_ 30 min vs. ZnCl_2_ 24 h.

On the basis of the data that the induction of CA1 LTP was more vulnerable to Zn-CQ injection than that of dentate gyrus LTP, object recognition memory was examined after intrahippocampal injection of Zn-CQ (9.8 nmol/rat) and ZnC1_2_ (9.8 nmol/rat) at 0.5 µl/min for 1 min. Intracellular levels of Zn^2+^ in the CA1 were increased 15 min after application of Zn-CQ and ZnCl_2_ to the hippocampal slices as described below. The local injection of Zn-CQ and ZnCl_2_ into the hippocampus is estimated to be similar situation to the in vitro application. Therefore the training of object recognition was performed 30 min after injection of vehicle, Zn-CQ and ZnC1_2_ into the CA1. There was no significant difference in exploratory time among vehicle-, ZnCQ- and ZnCl_2_-administered rats in the training trial. The time spent to explore the two identical objects was 78.7±6.3 s, 66.6±8.1 s, and 68.7±6.0 s in vehicle-, Zn-CQ- and ZnCl_2_-administered rats, respectively. The averaged recognition indices were 50.6±1.2%, 51.6±2.8%, and 50.3±2.7% in vehicle-, Zn-CQ-, and ZnCl_2_-administered rats, respectively. The recognition index in the test trial was significantly increased in vehicle-administered (control) rats than that in the training trial (62.2±4.4%, p<0.05, vs. training), but not in Zn-CQ-administered (46.9±2.9%, p<0.05, vs. control) and ZnCl_2_-administered (55.0±4.7%) rats (Fig. 4). On the other hand, the recognition index in the test trial was significantly increased in ZnCl_2_-administered (69.9±3.1%, p<0.01 vs. training; p<0.05 vs. ZnCl_2_ 30 min) rats when the training was performed 24 h after injection of ZnCl_2_ into the CA1.

In the case of their injections into the dentate gyrus, there was also no significant difference in exploratory time among vehicle-, Zn-CQ- and ZnCl_2_-administered rats in the training trial. The time spent to explore the two identical objects was 81.7±6.1 s, 74.3±7.1 s, and 81.4±5.8 s in the control, ZnCl_2_-administered, and Zn-CQ-administered rats, respectively. The averaged recognition indices were 52.2±7.9%, 47.1±1.8%, and 50.2±1.2% in vehicle-, Zn-CQ-, and ZnCl_2_-administered rats, respectively. The recognition index in the test trial was significantly increased in these three groups than that in the training trial (control, 66.6±2.8%; Zn-CQ, 58.5±3.4%; ZnCl_2_, 63.9±4.0%; p<0.05 vs. training) (Fig. 4).

**Figure 5:**
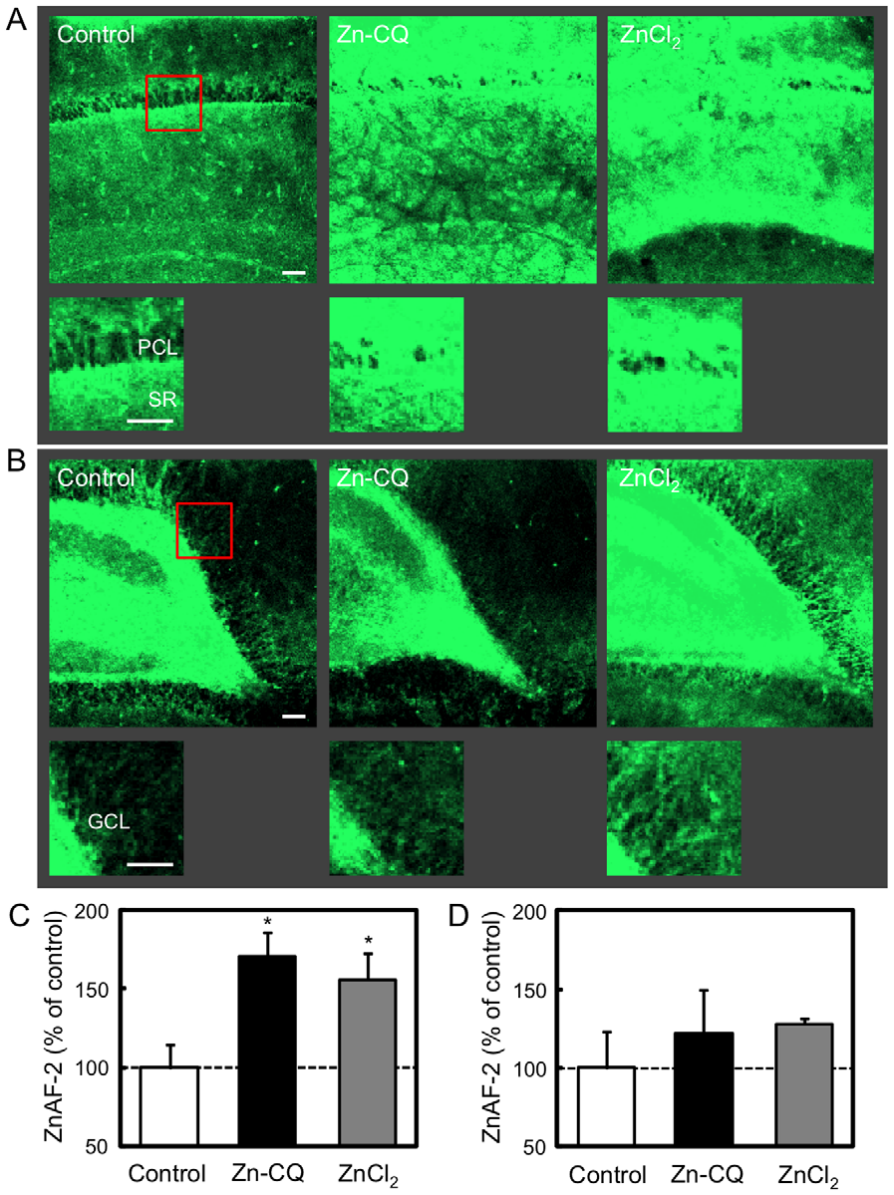
Increase in intracellular zinc after application of Zn-CQ and ZnCl_2_ to the hippocampus. Hippocampal slices stained with ZnAF-2DA were immersed in ACSF (control), Zn-CQ (4.4 µM) in ACSF, and ZnCl_2_ (4.4 µM) in ACSF for 15 min (n = 5). ZnAF-2 fluorescence was measured in the CA1 (A and C) and the dentate gyrus (B and D). Each lower panel shows the magnified area that is shown by a representative red square. Bars; 50 µm. PLC, pyramidal cell layer; SR, stratum radiatum. GCL, granule cell layer. (C and D). Each bar and line (mean ± SEM) represents the rate (%) of the intensity of ZnAF-2 fluorescence in hippocampal slices immersed with Zn-CQ or ZnCl_2_ to that immersed with ACSF, which was represented as 100%. *, p<0.05, vs. control (ACSF).

To evaluate intracellular levels of Zn^2+^ after intrahippocampal injection of vehicle, Zn-CQ, and ZnC1_2_, hippocampal slices stained with ZnAF-2DA were immersed in vehicle in ACSF, Zn-CQ (4.4 µM) in ACSF, and ZnC1_2_ (4.4 µM) in ACSF and ZnAF-2 fluorescence was measured 15 min after the immersion. It is estimated that extracellular levels of Zn^2+^ reach very low micromolar concentrations during LTP induction [23], [24]. The dose of zinc was used as a potential concentration of endogenous Zn^2+^ released under stressful conditions. Intracellular levels of Zn^2+^ were significantly increased in the pyramidal cell layer of the CA1 immersed with Zn-CQ and ZnC1_2_ (control, 100.0±14.3%; Zn-CQ, 171.0±14.9%; ZnCl_2_, 155.9±17.2%; p<0.05 vs. control) (Fig. 5A and C), but not in the granule cell layer of the dentate gyrus (control, 100.0±23.0%; Zn-CQ, 122.0±27.8%; ZnCl_2_, 127.6±4.0%) (Fig. 5B and D).

### Memory deficit by endogenous Zn^2+^ in the CA1

**Figure 6:**
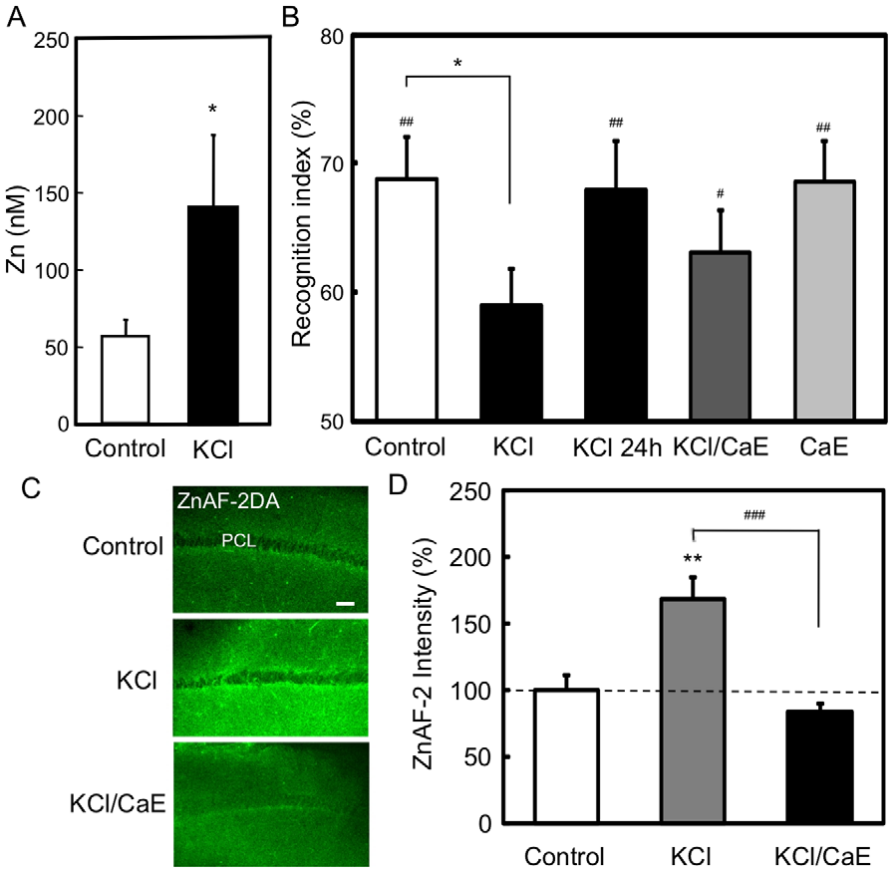
Transient object recognition memory deficit and increase in intracellular Zn^2+^ after CA1 excitation. (A) The hippocampus was perfused with ACSF for 60 min to determine the basal concentration of extracellular zinc, and perfused with 100 mM KCl in ACSF for 40 min to determine the change in the extracellular zinc concentration by neuronal depolarization. The perfusate was collected every 20 min. The control represents the mean of 3 samples before perfusion with 100 mM KCl. The 100 mM KCl represents the mean of 2 samples during perfusion with 100 mM KCl. Each bar and line represents the mean ± SEM (n = 6). *, p<0.05, vs. control. (B) One week after implantation of guide cannulae as described in the method section, rats were placed for 10 min into an open field. Twenty-four hours after the open field exploration, vehicle (control, n = 9), 100 mM KCl in vehicle (n = 15), 100 mM KCl+1 mM CaEDTA in vehicle (n = 8), and 1 mM CaEDTA in vehicle (n = 7) were bilaterally injected via injection cannulae into the hippocampal CA1 of the rats at the rate of 0.5 µl/min for 2 min. Thirty minutes after injection, rats were trained for 5 min and then tested for 3 min in the novel object recognition task 1 h after training. In the case of 100 mM KCl injection into the CA1, 24 h after injection, rats were subjected to the test in the same manner. Each bar and line represents the mean ± SEM. ^#^, p<0.05, ^##^, p<0.01, vs. training; *, p<0.05, vs. control. (C) Hippocampal slices were prepared from rats, stained with ZnAF-2DA and immersed with ACSF (control,), 100 mM KCl and 100 mM KCl containing 1 mM CaEDTA for 15 min (n = 8). PLC, pyramidal cell layer. (D). Each bar and line (mean ± SEM) represents the rate (%) of the intensity of ZnAF-2 fluorescence in hippocampal slices immersed with 100 mM KCl or 100 mM KCl containing 1 mM CaEDTA to that immersed with ACSF, which was represented as 100%. **, p<0.01, vs. control; ^###^, p<0.001, vs. KCl.

It was confirmed that the basal level of extracellular zinc was significantly increased by perfusion with 100 mM KCl in the hippocampus (basal (control), 57.9±9.1 nM; KC1, 140.0±47.1; p<0.05 vs. basal) (Fig. 6A). To check the action of excess release of endogenous Zn^2+^ in object recognition memory, 100 mM KC1 in saline, 100 mM KCl and 1 mM CaEDTA, a membrane-impermeable zinc chelator, in saline, and 1 mM CaEDTA in saline (1 µl) were injected into the CA1 at the rate of 0.5 µl/min for 2 min. Because extracellular levels of Zn^2+^ in the hippocampus were increased immediately after in vivo stimulation with KCl (Fig. 6A) and intracellular levels of Zn^2+^ in the CA1 were increased 15 min after in vitro immersion with KCl (Fig. 6D) as described below, the training of object recognition was performed 30 min after the injections. There was no significant difference in exploratory time among vehicle-, KCl-, KCl/CaEDTA-, and CaEDTA-administered rats in the training trial. The time spent to explore the two identical objects was 98.2±11.1 s, 102.3±10.4 s, 102.2±7.5 s, and 113.8±12.3 s in vehicle-, KCl-, KCl/CaEDTA-, and CaEDTA-administered rats, respectively. The averaged recognition indices were 50.8±1.4%, 52.7±1.4%, 49.3±2.0%, and 49.9±2.6% in vehicle-, KCl-, KCl/CaEDTA-, and CaEDTA-administered rats, respectively. The recognition index in the test trial was not significantly increased in KCl-administered rats, whereas it was significantly increased by co-administration of CaEDTA (control, 68.9±3.2%, p<0.01 vs. training; KCl, 59.0±2.9%, p<0.05 vs. control; KCl/CaEDTA, 63.2±3.2%, p<0.05 vs. training; CaEDTA, 68.7±3.1%, p<0.01 vs. training) (Fig. 6B). Furthermore, the recognition index in the test trial was significantly increased even in KCl-administered rats (67.6±5.9%, p<0.01 vs. training) when the training of object recognition was performed 24 h after the injection (Fig. 6B).

To confirm the increase in intracellular Zn^2+^ after stimulation with KCl and the effect of CaEDTA, hippocampal slices were immersed with 100 mM KCl and 100 mM KCl containing 1 mM CaEDTA. Intracellular Zn^2+^ measured with ZnAF-2 was significantly increased in the pyramidal cell layer of the CA1 by immersion with 100 mM KCl (control, 100.0±10.7%; KCl, 167.9±16.4%; p<0.01 vs. control), but not by 100 mM KCl containing 1 mM CaEDTA (KCl/CaEDTA, 83.3±5.9%) (Fig. 6C and D).

## Discussion

Chelatable zinc predominantly exists in the synaptic vesicles and is undetectable in the brains of mice with targeted disruption of the ZnT3 gene [33]. This evidence is obtained from the dramatic reduction in the intensity of Timm's stain in the brain. Chelatable zinc released into the synaptic cleft is estimated to serve in free form (Zn^2+^) and is immediately taken up into presynaptic and postsynaptic neurons during synaptic excitation. The basal Zn^2+^ concentrations are extremely low in both the extracellular (∼10^−8^ M) and intracellular (cytosolic) (<10^−9^ M) compartments [34], [35] and the increase in Zn^2+^ concentrations in both compartments serves for signaling [23], [36], [37]. However, the extracellular and intracellular concentrations of Zn^2+^ reached during and after synaptic excitation are obscure. Other organelles such as the mitochondria and the endoplasmic reticulum including the cytoplasm may also participate in the increase in cytosolic Zn^2+^ [38], [39], [40]. If extracellular Zn^2+^ reaches very low micromolar concentrations during synaptic excitation, it is possible that cytosolic Zn^2+^ transiently reaches 100 times more than the basal concentrations. Furthermore, cytosolic Zn^2+^ potentially reach submicromolar concentrations (−log[Zn^2+^]_“free”_<6) under pathological conditions [41], which participates in glutamate excitotoxicity and is involved in neurological diseases such as Alzheimer's disease [24]. Thus, it is necessary to study the relationship between a moderate increase in cytosolic Zn^2+^ and memory processing. Cytosolic Zn^2+^ might be moderately increased under stressful circumstances, followed by transient disturbance of synaptic plasticity such as LTP and memory processing [25], [26]. Exposure to acute stress elicits excess of extracellular glutamate in the hippocampus, followed by the disturbance of LTP induction and memory processing [42]. In the present study, the effect of the transient increase in Zn^2+^ delivered with Zn-CQ was examined focused on hippocampal LTP induction and object recognition memory.

Intracellular levels of Zn^2+^ measured with ZnAF-2DA were transiently increased in the CA1 pyramidal cell layer 2 h after i.p. injection of Zn-CQ (9.8 µmol/kg). ZnAF-2DA, diacetylated form of ZnAF-2, is taken up into cells and hydrolyzed to ZnAF-2, which cannot permeate the cell membrane [43]. ZnAF-2 has a low K_d_ value of 2.7×10^−9^ M for zinc and can measure Zn^2+^, which originates in Zn-CQ. CA1 LTP induced with 100-Hz tetanus was transiently attenuated in hippocampal slices prepared 2 h after Zn-CQ injection and restored in hippocampal slices prepared 24 h after injection. CA1 LTP induced with 100-Hz tetanus is significantly attenuated in hippocampal slices pre-perfused with 100 µM ZnCl_2_ and this attenuation is restored by perfusion with 10 µM 6-cyano-7-nitroquinoxaline-2,3-dione (CNQX), an AMPA/kainate receptor antagonist, which reduces Zn^2+^ influx into hippocampal cells, prior to the zinc pre-perfusion, suggesting that the increased influx of Zn^2+^ into hippocampal cells via AMPA/kainate receptor activation is an event to attenuate subsequent induction of CA1 LTP [44]. Therefore, it is likely that the transient increase in cytosolic Zn^2+^ in CA1 pyramidal neurons after Zn-CQ injection reversibly attenuates CA1 LTP induction. Zn^2+^ modifies calcium/calmodulin-dependent protein kinase II (CAMKII) activity that plays a key role for the induction of CA1 LTP [45], [46], [47]; Zn^2+^ can directly activate CaMKII, inhibits Ca^2+^/calmodulin binding to CAMKII and inactivates substrate phosphorylation activity of CaMKII at high concentrations [48]. Therefore, it is possible that the increased influx of Zn^2+^ modifies CaMKII activity, followed by the attenuation of CA1 LTP, although Zn^2+^ might also interact with other functional molecules to be involved in LTP induction.

Two hours after Zn-CQ injection, no significant difference in exploratory time between the control and Zn-CQ-administered rats in the training of object recognition suggests that the training was successfully performed in Zn-CQ-administered rats. However, object recognition memory deficit was observed in Zn-CQ-administered rats. To pursue the idea that the transient attenuation of CA1 LTP is linked to object recognition memory deficit, furthermore, hippocampal LTP was recorded in vivo. The induction of CA1 LTP was also attenuated 2 h after Zn-CQ injection, whereas that of dentate gyrus LTP was not attenuated, suggesting that the induction of CA1 LTP is more vulnerable to exposure to Zn-CQ than that of dentate gyrus LTP. The injection of Zn-CQ or ZnCl_2_ into the CA1 also elicited object recognition memory deficit, whereas the injection into the dentate gyrus had no significant effect on object recognition memory. The significant increase in Zn^2+^ in the CA1 pyramidal cell layer, unlike the dentate granule cell layer, in hippocanpal slices immersed with Zn-CQ or ZnC1_2_, suggesting that object recognition memory deficit is linked to the preferential increase in intracellular Zn^2+^ and/or the preferential vulnerability to Zn^2+^ in CA1 pyramidal neurons. It has been reported that calcium-permeable AMPA/kainate receptors elicits Zn^2+^ accumulation and neuronal loss in the CA1 after forebrain ischemia [49], [50], [51], [52]. Thus, calcium-permeable AMPA/kainate receptors may be involved in the increase in cytosolic Zn^2+^ in CA1 pyramidal neurons, followed by object recognition memory deficit.

Furthermore, the action of endogenous Zn^2+^ was examined after injection of 100 mM KCl into the hippocampus, which transiently increases the extracellular concentrations of glutamate and zinc in the hippocampus [53]. When 100 mM KCl (1 µl) was injected into the CA1, object recognition memory deficit was transiently observed. The memory deficit was ameliorated by the co-injection of 1 mM CaEDTA, a membrane-impermeable zinc chelator, which completely blocks cellular zinc uptake during synaptic excitation in the CA1 [37]. When KCl-induced increase in intracellular Zn^2+^ and the effect of CaEDTA were checked in hippocampal slices, intracellular Zn^2+^ was significantly increased in the pyramidal cell layer of the CA1 15 min after immersion with KCl, but not with KCl containing CaEDTA. It was confirmed that CaEDTA block the increase in intracellular Zn^2+^ during CA1 excitation. Therefore, the transient increase in zinc release from zincergic neurons in the CA1 may accumulate Zn^2+^ in CA1 pyramidal neurons and participate in object recognition memory deficit.

In conclusion, the present study indicates that transient dyshomeostasis of Zn^2+^ in CA1 pyramidal neurons reversibly impairs object recognition memory. Further investigation on dyshomeostasis of Zn^2+^ under stressful circumstances is necessary to understand not only stress-mediated impairment of learning and memory but also stress-mediated hippocampal dysfunction.

## Materials and Methods

### Animals and chemicals

Male Wistar rats (6 weeks old) were purchased from Japan SLC (Hamamatsu, Japan). Rats were housed under the standard laboratory conditions (23±1°C, 55±5% humidity) and had access to tap water and food ad libitum. All experiments were performed in accordance with the Guidelines for the Care and Use of Laboratory Animals of the University of Shizuoka that refer to American Association for Laboratory Animals Science and the guidelines laid down by the NIH (*NIH Guide for the Care and Use of Laboratory Animals*) in the USA. The Animal Experiment Committee of University of Shizuoka approved all protocols for animal experiments (#22-037).

To prepare Zn-CQ for i.p. and intrahippocampal injections, CQ was dissolved in 20% dimethyl sulfoxide (DMSO) in olive oil and DMSO, respectively, and ZnCl_2_ was added to each solution (CQ ∶ Zn = 2 ∶ 1). ZnAF-2DA, a membrane-permeable zinc indicator, was kindly supplied from Sekisui Medical Co., LTD (Tokai, Japan), dissolved in DMSO, and then diluted to artificial cerebrospinal fluid (ACSF) containing 119 mM NaCl, 2.5 mM KCl, 1.3 mM MgSO_4_, 1.0 mM NaH_2_PO_4_, 2.5 mM CaCl_2_, 26.2 mM NaHCO_3_, and 11 mM D-glucose (pH 7.3).

### Hippocampal slice preparation

Rats were anesthetized with ether and decapitated. The brain was quickly removed and immersed in ice-cold ACSF. Transverse hippocampal slices (400 µm) were prepared using a vibratome ZERO-1 (Dosaka Kyoto, Japan) in an ice-cold ACSF. Slices were then maintained in a holding chamber at room temperature for at least 1 h. All solutions used in the experiments were continuously bubbled with 95% O_2_ and 5% CO_2_.

### Zinc imaging

The hippocampal slices were immersed in 10 µM ZnAF-2DA in ACSF for 30 min and then washed out with ACSF for 1 h. For intracellular zinc imaging, the hippocampal slices were transferred to a recording chamber filled with 2 ml ACSF. The fluorescence of ZnAF-2 (excitation, 488 nm; monitoring, 505–530 nm) was measured in the hippocampus by using a confocal laser-scanning microscopic system LSM 510 (Carl Zeiss), equipped with the inverted microscope (Axiovert 200M, Carl Zeiss). Region of interest was set in the pyramidal cell layer of the CA1 and the granule cell layer of the dentate gyrus.

### In vitro CA1 LTP

The hippocampal slices were transferred to a recording chamber and submerged beneath a continuously superfusing ACSF, which was maintained at 26–27°C. The Schaffer collateral/commissural-CA1 pyramidal neuron responses were induced by stimulation of the Schaffer collateral/commissural pathway with a bipolar tungsten electrode. Extracellular recording was obtained by using a glass micropipette filled with 3 M NaCl (2–10 MΩ). The recording electrode was placed along the trajectory of Schaffer collateral/commissural pathway. Test stimuli (0.033 Hz, 200 µsec/pulse) were delivered to the Schaffer collateral/commissural pathway every 30 s. The stimulus intensity was set to produce approximately 40% (100–200 µA) of the maximum field excitatory postsynaptic potential (fEPSP). At the beginning of the experiments, the physiological state of the slices was tested by verifying the existence of paired-pulse facilitation, which was induced by application of paired pulses separated by 40 ms. CA1 LTP was induced by tetanic stimuli at 100 Hz for 1 s. Field EPSP amplitudes were averaged over 180-second intervals and expressed as percentages of the mean fEPSP amplitude measured during the 20-min baseline period perfused with ACSF, which was expressed as 100%.

### In vivo CA1 and Dentate gyrus LTP

CA1 and dentate gyrus LTP were recorded under anesthesia [54]. Male rats were anesthetized with chloral hydrate (400 mg/kg) and placed in a stereotaxic apparatus. A bipolar stimulating electrode and a monopolar recording electrode made of tungsten wire were positioned stereotaxically so as to selectively stimulate the Schaffer collateral/commissural pathway and the perforant pathway, while recording in the CA1 and the dentate gyrus, respectively [55]. The electrode stimulating the Schaffer collateral/commissural pathway was implanted 4.8 mm posterior to the bregma, 3.8 mm lateral, 2.9 mm inferior to the dura at an angle of 10 degrees toward the anterior-posterior plane. A recording electrode in the CA1 was implanted ipsilaterally 3.3 mm posterior to the bregma, 2.2 mm lateral and 1.9 mm inferior to the dura. The electrode stimulating the perforant pathway was implanted 8.0 mm posterior to the bregma, 4.5 mm lateral, 3.0–3.5 mm inferior to the dura. A recording electrode in the dentate gyrus was implanted ipsilaterally 4.0 mm posterior to the bregma, 2.3 mm lateral and 3.0–3.5 mm inferior to the dura. All the stimuli were biphasic square wave pulses (200 µs width) and their intensities were set at the current that evoked 40% of the maximum population spike (PS) amplitude. Test stimuli (0.05 Hz) were delivered at 20 s intervals to monitor PS.

At the beginning of the experiments, input/output curves were generated by systematic variation of the stimulus current (0.1–0.5 mA for the CA1 and 0.1–1.0 mA for the dentate gyrus) to evaluate synaptic potency. After stable baseline recording for at least 30 min, LTP was induced by delivery of high-frequency stimulation (HFS; 10 trains of 20 pulses at 200 Hz separated by 1 s). PS amplitude (test frequency: 0.05 Hz) were averaged over 120-second intervals and expressed as percentages of the mean PS amplitude measured during the 30-min baseline period perfused with ACSF prior to LTP induction, which was expressed as 100%.

### Object recognition memory

Rats were placed for 10 min into an open field, which was a 70×60 cm arena surrounded by 70 cm high walls, made of a black-colored plastic. Twenty-four hours after open field exploration, rats were trained and tested in a novel object recognition task as reported previously [56]. Training in the object recognition task took place in the same area used for the open field exploration. The open field exploration was thus used as a context habituation trial for the recognition memory task. The object recognition test requires that the rats recall which of two earthenware objects they had been previously familiarized with. Twenty-four hours after arena exploration, training was conducted by placing individual rats into the field, in which two identical objects (objects A1 and A2; sake bottle) were positioned in two adjacent corners, 15 cm from the walls. Rats were left to explore the objects for 5 min. Rats were not used for the test when the total of the object exploration time was less than 20 s. In the test given 1 h after training, the rats explored the open field for 3 min in the presence of one familiar (A) and one novel (B; cup) object. All objects presented similar textures, colors and sizes, but distinctive shapes. A recognition index calculated for each rat was expressed by the ratio T_B_/(T_A_+T_B_) [T_A_ = time spent to explore the familiar object A; T_B_ = time spent to explore the novel object B]. Between trials the objects were washed with 70% ethanol solution. Exploration was defined as sniffing or touching the object with the nose and/or forepaws. Sitting on the object was not considered as exploration. To check the preference for the novel object in the test, the novel object (cup) was changed with the familiar object (sake bottle). No preference for the objects was confirmed in the initial experiment.

### Implantation of guide cannulae into the hippocampus for local injection

The rats were anesthetized with chloral hydrate (400 mg/kg) and individually placed in a stereotaxic apparatus. The skull was exposed, a burr hole was drilled, and guide cannulae (CXG-6, EICOM Co., Kyoto, Japan) was implanted into the CA1 (3.3 mm posterior to the bregma, ±1.6 mm lateral, 2.3 mm inferior to the dura) or the dentate gyrus (3.8 mm posterior to the bregma, ±1.6 mm lateral, 3.2 mm inferior to the dura) of the right and left hippocampi, based on the coordinate of the brain map [55]. The guide tube was secured with dental cement and screws. After the surgical operation, each rat was housed individually. One week later, injection cannulae (CXMI-6, EICOM Co.) were inserted via the guide cannulae to inject Zn-CQ and ZnCl_2_ into the CA1 or the dentate gyrus under conscious condition.

### In vivo microdialysis

The rats were anesthetized with chloral hydrate (400 mg/kg) and individually placed in a stereotaxic apparatus. The skull was exposed, a burr hole was drilled, and a guide tube (CMA Microdialysis, Solna, Sweden) was implanted into the right hippocampus (5.6 mm posterior to the bregma, 4.6 mm lateral, 3.1 mm inferior to the dura), based on the coordinate of the brain map [55]. The guide tube was secured with dental cement and screws. After the surgical operation, each rat was housed individually. Forty-eight hours after implantation of the guide tube, a microdialysis probe (3-mm membrane CMA 12 probe, CMA Microdialysis) was inserted into the hippocampus of chloral hydrate-anesthetized rats through the guide tube. The hippocampus was preperfused with ACSF (127 mM NaCl, 2.5 mM KCl, 1.3 mM CaCl_2_, 0.9 mM MgCl_2_, 1.2 mM Na_2_HPO_4_, 21 mM NaHCO_3_ and 3.4 mM D-glucose, pH 7.3) at 5.0 µl/min for 60 min to stabilize the region, perfused for 60 min in the same manner to determine the basal concentration of extracellular zinc, and perfused with 100 mM KCl in ACSF at 5.0 µl/min for 40 min to determine the change in the extracellular zinc concentration by neuronal depolarization. The perfusate was collected every 20 min.

### Extracellular zinc concentration

The perfusate samples (50 µl) were diluted with 2% nitric acid (100 µl). Analysis of the samples in triplicate was conducted using a flameless atomic absorption spectrophotometer (Shimadzu AA6800F, Kyoto, Japan). Zinc concentration in the samples was determined by a calibration curve prepared from zinc standard solution (Woko Pure Chemical Industries, Ltd., Osaka, Japan).

### Statistical analysis

Grouped data are expressed as the mean ± SEM. Student's *t*-test was used for comparison of the means of paired or unpaired data. For multiple comparisons, two-way ANOVA was used as indicated (the statistical software, GraphPad Prism 5).
